# Identification of a Subtype-Selective Allosteric Inhibitor of GluN1/GluN3 NMDA Receptors

**DOI:** 10.3389/fphar.2022.888308

**Published:** 2022-06-09

**Authors:** Yue Zeng, Yueming Zheng, Tongtong Zhang, Fei Ye, Li Zhan, Zengwei Kou, Shujia Zhu, Zhaobing Gao

**Affiliations:** ^1^ Center for Neurological and Psychiatric Research and Drug Discovery, Shanghai Institute of Materia Medica, Chinese Academy of Sciences, Shanghai, China; ^2^ College of Pharmacy, University of Chinese Academy of Sciences, Beijing, China; ^3^ State Key Laboratory of Neuroscience, CAS Center for Excellence in Brain Science and Intelligence Technology, Institute of Neuroscience, Chinese Academy of Sciences, Shanghai, China; ^4^ College of Life Sciences and Medicine, Zhejiang Sci-Tech University, Hangzhou, China; ^5^ Zhongshan Institute of Drug Discovery, Institution for Drug Discovery Innovation, Chinese Academy of Sciences, Zhongshan, China

**Keywords:** N-methyl-D-aspartate (NMDA) receptors, GluN3 subunits, WZB117, allosteric modulator, ion channels, drug discovery

## Abstract

N-methyl-D-aspartate receptors (NMDARs) are Ca^2+^-permeable ionotropic glutamate receptors (iGluRs) in the central nervous system and play important roles in neuronal development and synaptic plasticity. Conventional NMDARs, which typically comprise GluN1 and GluN2 subunits, have different biophysical properties than GluN3-containing NMDARs: GluN3-containing NMDARs have smaller unitary conductance, less Ca^2+^-permeability and lower Mg^2+^-sensitivity than those of conventional NMDARs. However, there are very few specific modulators for GluN3-containing NMDARs. Here, we developed a cell-based high-throughput calcium assay and identified 3-fluoro-1,2-phenylene bis (3-hydroxybenzoate) (WZB117) as a relatively selective inhibitor of GluN1/GluN3 receptors. The IC_50_ value of WZB117 on GluN1/GluN3A receptors expressed in HEK-293 cells was 1.15 ± 0.34 μM. Consistently, WZB117 exhibited strong inhibitory activity against glycine-induced currents in the presence of CGP-78608 but only slightly affected the NMDA-, KA- and AMPA-induced currents in the acutely isolated rat hippocampal neurons. Among the four types of endogenous currents, only the first one is primarily mediated by GluN1/GluN3 receptors. Mechanistic studies showed that WZB117 inhibited the GluN1/GluN3A receptors in a glycine-, voltage- and pH-independent manner, suggesting it is an allosteric modulator. Site-directed mutagenesis and chimera construction further revealed that WZB117 may act on the GluN3A pre-M1 region with key determinants different from those of previously identified modulators. Together, our study developed an efficient method to discover modulators of GluN3-containing NMDARs and characterized WZB117 as a novel allosteric inhibitor of GluN1/GluN3 receptors.

## Introduction

N-methyl-D-aspartate receptors (NMDARs) are glutamate-gated ion channels that play pivotal roles in neuronal development and synaptic plasticity in the central nervous system (Traynelis et al., 2010; Paoletti et al., 2013; Hansen et al., 2018). Seven NMDAR subunits have been identified, including GluN1, GluN2 (2A-2D) and GluN3 (3A-3B) subunits. Conventional NMDARs are heterotetramers comprising two glycine-binding GluN1 subunits and two glutamate-binding GluN2 (2A-2D) subunits, with high Ca^2+^-permeability and voltage-dependent Mg^2+^ blockage (Ulbrich and Isacoff, 2007; Paoletti et al., 2013; Karakas and Furukawa, 2014). Over the past 3 decades, conventional NMDARs have been intensively studied as potential therapeutic targets for various psychiatric or neurological disorders (Hansen et al., 2021).

Much less is known about GluN3-containing NMDARs (hereafter GluN3-NMDARs) than about conventional NMDARs (Crawley et al., 2022). Coassembly with GluN3 (3A-3B) subunits dramatically changes the biophysical properties of NMDARs by promoting smaller unitary conductance, less Ca^2+^-permeability and lower Mg^2+^-sensitivity. Thus, GluN3A and GluN3B have been proposed to be dominant negative subunits of NMDARs (Das et al., 1998; Perez-Otano et al., 2001; Chatterton et al., 2002). Under physiological conditions, GluN3A expression is maintained in a narrow time window in early postnatal development and remains at a low level in adults (Ciabarra et al., 1995; Sucher et al., 1995; Sasaki et al., 2002; Wong et al., 2002). Multiple studies have reported that GluN3A may regulate synaptic maturation and pruning (Perez-Otano et al., 2006; Roberts et al., 2009; Fiuza et al., 2013; Kehoe et al., 2013; Kehoe et al., 2014). Dysfunction of GluN3A due to failure of downregulation or aberrant reactivation beyond the physiological time window is implicated in multiple neurological diseases, such as Huntington’s disease (Marco et al., 2013; Wesseling and Perez-Otano, 2015; Mahfooz et al., 2016; Marco et al., 2018), addiction (Yuan and Bellone, 2013; Yuan et al., 2013; Jin et al., 2014), and schizophrenia (Glantz and Lewis, 2000; Mueller and Meador-Woodruff, 2004; Takata et al., 2013). Different from that of GluN3A, the expression of GluN3B increases slowly throughout the developmental stages but appreciably in caudal areas (Chatterton et al., 2002). Limited studies have suggested that GluN3B may be involved in motor function, as the expression of GluN3B is restricted to the motor neurons of spinal cord and brainstem (Chatterton et al., 2002). The pharmacological and physiological functions of these two GluN3 subunits need to be further studied, especially regarding whether they can be drug targets for the treatment of neurological diseases, in which GluN3-specific modulators may serve as pharmacological tools.

In contrast to conventional NMDARs, GluN1/GluN3 receptors belong to a special class of NMDARs that only require glycine for activation, since the endogenous ligands are glycine for both GluN1 and GluN3 (Ciabarra et al., 1995; Sucher et al., 1995; Chatterton et al., 2002; Sasaki et al., 2002; Kehoe et al., 2014). However, the glycine-induced currents measured with conventional methods such as whole-cell patch-clamp recording of GluN1/GluN3 receptors are negligible, possibly due to their rapid desensitization caused by the preferential binding of glycine to the GluN1 subunit (Grand et al., 2018). Mutagenesis of the glycine-binding sites (GluN1^F484A/T518L^) (Kvist et al., 2013) or pre-incubation with CGP-78608, a competitive antagonist of GluN1 (Grand et al., 2018), can prevent desensitization, which has enabled the discovery of GluN3-specific modulators, including TK series compounds (TK13, TK30, TK80) and EU1180-438 (Kvist et al., 2013; Zhu et al., 2020). Although TK compounds are less active and relatively mediocre in selectivity, their discovery suggests that the glycine-binding pocket in the GluN3A subunit could be suitable for GluN3-specific pharmaceutical development, as reflected in a 650-fold higher affinity of glycine for GluN3A than for GluN1 (Yao and Mayer, 2006). EU1180-438 was reported as a negative allosteric modulator of GluN1/GluN3 receptors with high potency and selectivity acting on the Glu3A pre-M1 region, a linker between the ligand binding domains (LBDs) and the first transmembrane helix (M1) (Zhu et al., 2020). In short, pharmacological tools targeting GluN3 are still scarce. Thus, it is important to develop a more efficient method to identify novel and potent modulators targeting GluN3-NMDARs.

Here, we developed a cell-based high throughput calcium assay and identified multiple novel inhibitors of GluN3-NMDARs from 2560 compounds. Most of the compounds were obtained from the Approved Drug Library (originally from Topscience, MCE, ApexBio and Selleck), and the rest that are in clinical or preclinical stages were collected by our lab. These compounds generally have known and well-characterized biological activity, safety and bioavailability. Among the identified hits, WZB117 was characterized as an allosteric inhibitor with high selectivity for recombinant and native GluN1/GluN3 receptors.

## Material and Methods

### Molecular Biology

To establish GluN1/GluN3A stable cell lines, the human GluN1-1a (GenBank: NP_015566.1) and the rat GluN3A (GenBank: NP_612555.1) genes were subcloned into the pcDNA5-FRT-To and pcDNA3.1 vectors, respectively. An EGFP tag was added to the C-terminus of GluN1 or GluN3A using a T2A linker for identification of transfected cells during transient transfection. Point mutations were introduced by site-directed mutagenesis using PrimeSTAR (Takara). The human GluN2A (GenBank: NP_000824.1) gene was subcloned into the pcDNA3.1 vector. The chimeras GluN3A^670-673 MWPL/LEPF^ and GluN2A^550-553 LEPF/MWPL^ were generated using In-Fusion HD Cloning kits (Takara). All constructs were confirmed by DNA sequencing (Beijing Genomics Institute).

### Cell Culture and Transfection

The Flp-in T-Rex 293 cell lines (Invitrogen), stably expressing GluN1/GluN3A were grown in DMEM basal medium (Gibco) supplemented with 10% fetal bovine serum (FBS, Invitrogen), 15 μg/ml blasticidin S (Invitrogen), 50 μg/ml hygromycin B (Invitrogen) and 500 μg/ml G418 (Gibco). Expression in cell lines was induced with 1 μg/ml doxycycline (Invitrogen) 24–28 h prior to the experiments. Chinese hamster ovary (CHO) cells were grown in DMEM-F12 basal medium (Gibco) supplemented with 10% FBS. In the study on the action sites of WZB117, all whole-cell patch-clamp recordings were performed on CHO cells. The transfections were carried out using Lipofectamine 3000 transfection reagent (Thermo Fisher) according to the manufacturer’s instructions. All cells mentioned above were cultured in a 5% CO_2_ incubator at 37°C and passaged every other day at a ratio of 1:3.

### qRT-PCR

Total RNA was extracted from cells using TRIzol (Invitrogen) and reverse-transcribed into cDNA using PrimeScript RT Master Mix (Takara) following the manufacturer’s instructions. The quantifications were performed using the SYBR Green Master Mix kit (Yeasen) in the Fast Real-Time PCR system (Applied Biosystems 7500, CA, United States). The results were processed with the mean values of delta Ct and Standard Error of Mean (SEM). The primers used for quantification are as follows:

GluN1-F: 5′-CCA​GTC​AAG​AAG​GTG​ATC​TGC​AC-3′;

GluN1-R: 5′-TTC​ATG​GTC​CGT​GCC​AGC​TTG​A-3′;

GluN3A-F: 5′-GCA​TAG​TGC​GCC​ACG​AGT​T-3′;

GluN3A-R: 5′-GGT​CAG​GAT​TGA​GAC​AGT​GAC​AT-3′;

GAPDH-F: 5′-GTC​AAG​GCT​GAG​AAC​GGG​AA-3′;

GAPDH-R: 5′-AAA​TGA​GCC​CCA​GCC​TTC​TC-3′.

### Western Blot Analysis

Total protein was extracted with strong RIPA lysis buffer (Yeasen), quantified with the BCA protein quantification kit (Thermo Fisher) and separated by 8% SDS-PAGE electrophoresis, and then the membrane was incubated with primary antibodies against GluN3A (at a working concentration of 10 μg/ml) and GAPDH (Yeasen, at a working concentration of 1 μg/ml). The primary antibody against GluN3A was produced by our own lab. Briefly, GluN3A-NTD protein was used as an antigen to immunize Balb/c mice. Seropositive splenocytes detected by ELISA were fused with myeloma cells (SP2/0) to obtain hybridoma cells. Total RNA was extracted from hybridoma cells and reverse-transcribed into cDNA. The sequence of the heavy and light chains of the antibody were cloned and sequenced using multiple mixed primers (von Boehmer et al., 2016), and the antibody was expressed and purified in HEK-293T cells *in vitro*. The second antibody was peroxidase AffiniPure goat anti-mouse IgG (H + L) (Yeasen).

### High-Throughput Screening Assay

The HEK-293 cells stably expressing GluN1/GluN3A receptors were seeded at a density of 10,000 cells/well in the 384-well plates, and expression was induced with 1 μg/ml doxycycline for 24–28 h. Then the culture medium was removed, Fluo-4 (Invitrogen) was loaded and the cells were incubated in the 37°C incubator away from light for 1 h (Gee et al., 2000). The plates were gently washed twice with Ca^2+^-free buffer and then incubated with one of the 2560 compounds (from the Chinese National Compound Library) or DMSO (1% and 0.33%) for 20 min. A fixed concentration of 30 μM and dual concentrations of 10 μM and 30 μM were applied in the primary and secondary screening, respectively. Two replicates were set up for each condition. After incubating 30 μl of the tested compounds, 10 μl of CGP-78608 (Tocris, at a working concentration of 500 nM) was added and incubated for 3 min, and the cells were subsequently stimulated by 10 μl of glycine (Amresco, at a working concentration of 100 μM). A FDSS/μCell platform (Hamamatsu) was used to measure the fluorescence signals. The excitation (Ex) and emission (Em) wavelengths were set to 480 and 540 nm, respectively. The inhibitory effects of tested compounds were compared by the ratios of fluorescence (*F*) with (*F*_drug_) and without (*F*_control_) the compounds. The *F*_control_ value in these assays represented the value of basal Fluo-4 fluorescence plus glycine-stimulated fluorescence, and the *F*_drug_ indicated the total Fluo-4 fluorescence after treatment of the drugs. Both the *F*_drug_ and *F*_control_ values were taken at the peak. The Ca^2+^-free buffer used to dilute Fluo-4 and wash cell-plates contained (in mM): 140 NaCl, 5 KCl, 1 MgCl_2_, 10 glucose, 10 HEPES, and 0.5 EGTA. The pH was adjusted to 7.2 using NaOH. To dilute the compounds, 2 mM of free Ca^2+^ was added in addition to the other components of the buffer.

### Acutely Isolated Hippocampal Neuron Preparations

Hippocampal neurons were obtained from Sprague-Dawley rats (aged P7-14). Briefly, the isolated hippocampal tissues were cut into approximately 800 μM slices along the sagittal plane and were digested with 3 mg/ml proteinase (Sigma-Aldrich) at 32°C for 8 min. Then 1 mg/ml bovine serum albumin (Sangon Biotech) and 1 mg/ml trypsin inhibitor (Sigma-Aldrich) were used to stop the digestion. A few slices of the hippocampus were gently blown into single cells with the dissecting solution, then transferred to a dish and left to rest for 20 min before experiments. The dishes were preprocessed. Essentially, the bottom of the dish was covered with discarded cell suspension the day before the experiment and was washed and dried the next day, which prevented the neurons from sticking to the bottom too tightly. The dissecting solution contained (in mM): 82 Na_2_SO_4_, 30 K_2_SO_4_, 5 MgCl_2_, 1 Na pyruvate, 10 HEPES, and 20 glucose. The pH was adjusted to 7.2 using NaOH (Xie et al., 2020).

### Whole-Cell Patch-Clamp Recording

Whole-cell patch-clamp recordings were performed at room temperature (22–25°C) using an EPC-10 amplifier and Patch Master Software (HEKA). Pipettes were pulled from borosilicate glass capillaries (World Precision Instruments), and the resistances were 3–5 MΩ when filled with the intracellular solution. The extracellular solution contained (in mM): 140 NaCl, 2.8 KCl, 1 CaCl_2_, 10 HEPES and 20 sucrose (290–300 mOsm), pH adjusted to 7.3 using NaOH. The pipette solution contained (in mM): 115 CsF, 10 CsCl, 10 HEPES and 10 BAPTA (280–290 mOsm), pH adjusted to 7.2 using CsOH (Grand et al., 2018). When recording in hippocampal neurons, the extracellular solution contained (in mM): 140 NaCl, 5 KCl, 1 CaCl_2_, 1.25 MgCl_2_, 10 glucose, and 10 HEPES, and the pH was adjusted to 7.4 using NaOH, and the pipette solution contained (in mM): 140 KCl, 1 MgCl_2_, 10 EGTA, and 10 HEPES, and the pH was adjusted to 7.2 using KOH (Xie et al., 2020). Drug-containing solutions were applied through an RSC-200 Rapid Solution Changer with a 9-tube head (BioLogic Co., France), in which the cell is positioned close to the output of a capillary. The solution, containing either one drug or a combination of different drugs, to be assayed flows out of the capillary at a moderate flow rate by gravity. The lifted cell, exposed to this stream, rapidly equilibrates in the perfusion solution. Whole-cell patch-clamp recordings in heterologous cells and hippocampal neurons were performed at a holding potential of −60 mV. Currents were sampled at 10 kHz and low pass filtered at 2.9 kHz. To record the native GluN1/GluN3 receptor-mediated currents, the extracellular solution was supplemented with 10 μM bicuculline (Sigma-Aldrich), 2 μM NBQX (Topscience), 100 μM D-APV (MCE), and 50 μM strychnine (UHN Shanghai) to block γ-aminobutyric acid type A (GABA_A_), α-amino-3-hydroxy-5-methylisoxazole-4-propionate (AMPA), GluN1/GluN2 NMDA and glycine receptors, respectively. To record the native GluN2-NMDARs-mediated currents, 10 μM bicuculline and 2 μM NBQX were added into the extracellular solution to block GABA_A_ receptors and AMPA receptors, respectively (Grand et al., 2018; Otsu et al., 2019; Zhu et al., 2020).

### Two-Electrode Voltage-Clamp Recordings

Two-electrode voltage-clamp (TEVC) recordings were performed on *Xenopus* oocytes expressing various subtypes of recombinant NMDARs. Each oocyte was injected with a mixture of 0.5–1.0 ng cDNAs or cRNAs encoding wild-type (WT) or mutant GluN1 (GluN1-1a or GluN1-4a) and GluN2 (2A-2D) or GluN3 (3A-3B) at a ratio of 1:1. The mutant GluN1-4a ^F484A/T518L^ was injected together with GluN3B to boost the currents of GluN1/GluN3B receptors, and WT GluN1-1a was used unless otherwise stated. The cRNAs of GluN1-4a, GluN3A and GluN3B were transcribed from corresponding DNAs using the mMESSAGE mMACHINE T7 Ultra kit (Life Technologies) according to the manufacturer’s instructions, and the others not specified were cDNAs. TEVC recording was performed 24–72 h post injection in extracellular solution containing (in mM): 100 NaCl, 0.3 BaCl_2_, 2.5 KCl, 0.01 DTPA and 5 HEPES, and the pH was adjusted to 7.3 using NaOH. All recordings were carried out at a holding potential of −60 mV.

### Molecular Modeling

The structural model of GluN1/GluN3A receptor was generated using Modeller 9.24 (Webb and Sali, 2016) based on two templates, the diheteromeric GluN1/GluN2B receptor structure (PDB ID: 6WHS) (Chou et al., 2020) and the truncated GluN3A-LBD structure (PDB ID: 2RC7) (Yao et al., 2008). Then, the structure was refined using the Protein Preparation Wizard Workflow embedded in the Maestro 9.0, and all the parameters were set as the default. The potential binding pockets around the pre-M1 region of GluN1/GluN3A receptors were predicted using the Fpocket program (Le Guilloux et al., 2009). Afterward, the program Glide (Friesner et al., 2004) integrated in Maestro was employed to perform molecular docking. The residues in the range of 10 Å around the residue W671 were defined as the binding sites. The compound WZB117 was prepared through LigPrep provided by Maestro to generate protonation states with Epik (Shelley et al., 2007) with a pH value of 7.0 ± 2.0. The Glide XP mode was used to dock the prepared ligand into the defined docking grid of GluN1/GluN3A receptors.

### Statistical Analysis

Patch-clamp data were processed using Clampfit 10.4 (Molecular Device, Sunnyvale, CA, United States) and analyzed with GraphPad Prism 5.0 (GraphPad Software, San Diego, CA, United States). Concentration-response curves were fitted using the following 3-parameter Hill equation: Y=Bottom + (Top-Bottom)/(1 + 10^(X-LogIC_50_)), where Top and Bottom, respectively, represent the channel’s maximum and minimum responses to the compounds, X is the value of the logarithm of the concentration, Y is the *I*_drug_/*I*_control_ value, and IC_50_ is the drug concentration producing a half-maximum response. All results are given as the mean ± SEM. Statistical analyses were performed using Student’s t-test or two-way ANOVA. Asterisks (*) indicate statistically significant differences from the control group (*p < 0.05, **p < 0.01 and ***p < 0.001).

## Results

### Establishment of a HTS Method for GluN1/GluN3A Receptors

**FIGURE 1:**
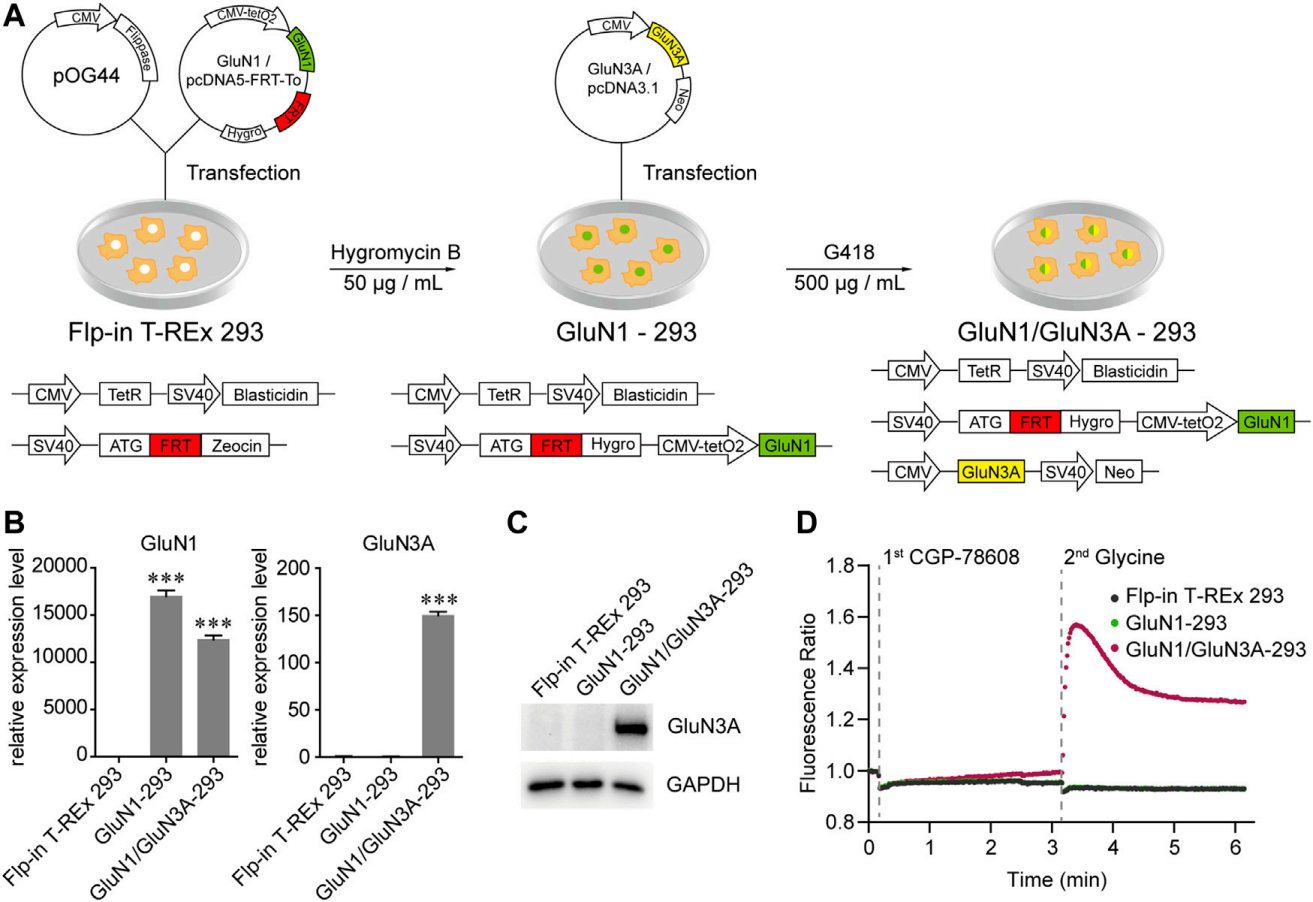
Generation and functional verification of GluN1/GluN3A Flp-in T-REx 293 stable cell lines. **(A)** Schematic graph showing the generation of GluN1/GluN3A Flp-in T-REx 293 stable cell lines. **(B and C)** Expression levels of GluN1 and GluN3A in the GluN1/GluN3A stable cell lines verified by qRT-PCR **(B)** and Western Blot **(C)**. **(D)** GluN1/GluN3A stable cell lines formed functional ion channels. When the channels were activated by CGP-78608 and glycine, Ca^2+^ flowed into cells and bound to Fluo-4, resulting in a fluorescence signal detected by a FDSS/μCell platform. The basal fluorescence value was automatically normalized to 1 in the FDSS/μCell platform, so the real-time fluorescence response was displayed as a fluorescence ratio. Significance was tested using one-way ANOVA; ∗*p* < 0.05, ∗∗*p* < 0.01 and ∗∗∗*p* < 0.001.

To discover GluN3A-specific modulators, we have developed a cell-based HTS method by measuring Ca^2+^ influx using Fluo-4 as fluorescent indicator. First, a cell line stably expressing GluN1/GluN3A receptors was established using the Flp-in T-REx system (Figure 1A). To induce GluN1/GluN3A expression, 1 μg/ml doxycycline was added into the culture medium 24–28 h before the experiments. The GluN1/GluN3A Flp-in T-REx 293 stable cell lines remained healthy despite the induction. Unlike in the host cells, qRT-PCR (Figure 1B) and Western blot assays (Figure 1C) showed that both GluN1 and GluN3A were well expressed in the GluN1/GluN3A stable cell lines with induction. As illustrated in Figure 1D, after administration of CGP-78608 and glycine, only the GluN1/GluN3A stable cell line yielded fluorescence signal, whereas neither GluN1 stable cell lines nor host cells did. These data showed that a functional HTS method was successfully established.

### Identification of Modulators for GluN1/GluN3A Receptors

**FIGURE 2:**
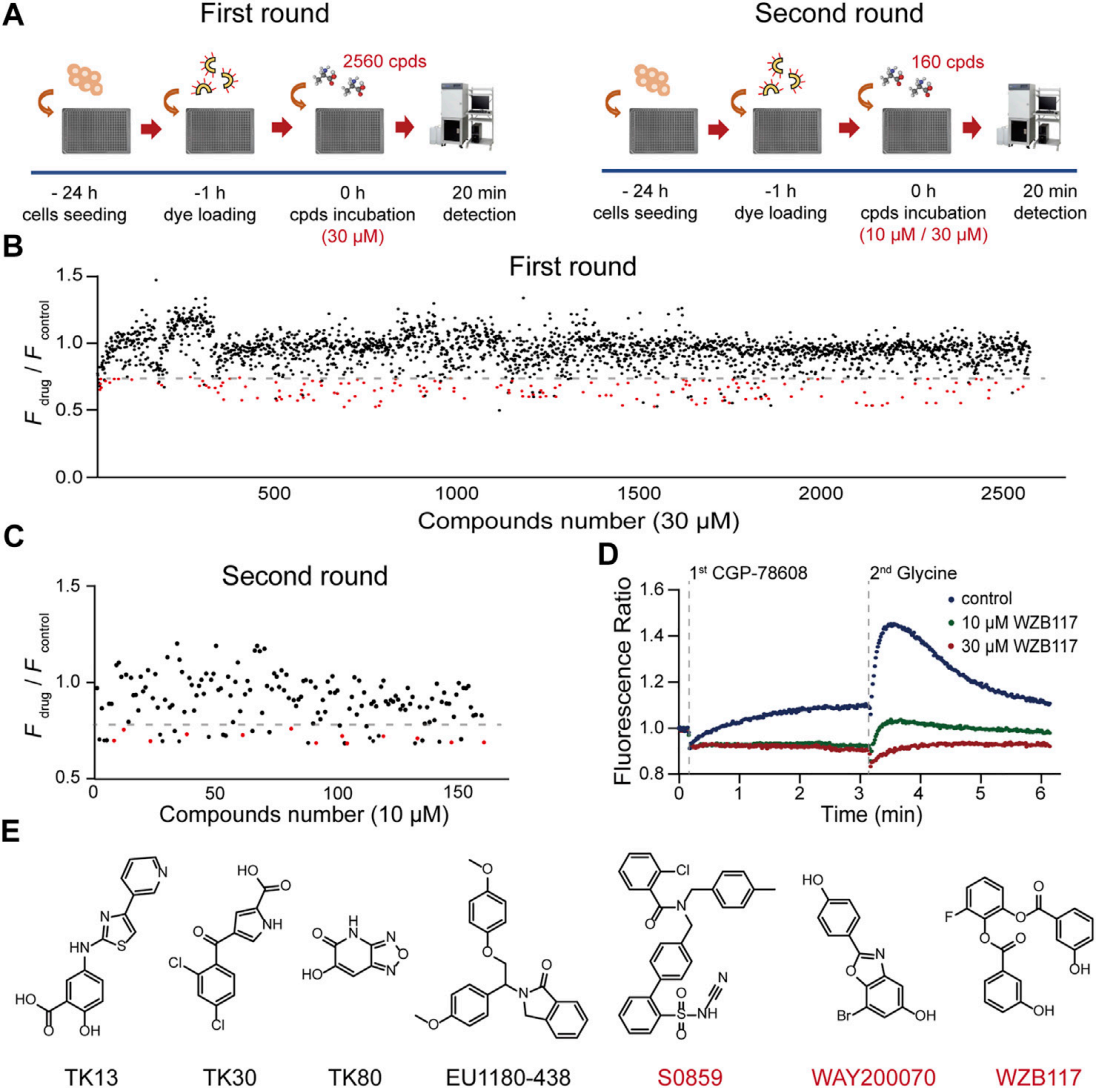
HTS for GluN1/GluN3A modulators. **(A)** Flow chart of the HTS. **(B)** Overview of the first round results. The Ca^2+^ fluorescence signal ratio (*F*_drug_/*F*_control_) of all compounds (2560) is represented by a circle. Compounds (30 μM) with a ratio less than 0.75 (the dashed line) were considered as the pre-hits and are displayed as red circles. Data represent the mean of the duplicate results. **(C)** Discovery of hits. A total of 160 pre-hits were selected for the second round. Compounds (10 μM) with a ratio less than 0.75 (the dashed line) and showing a dose-dependent inhibitory activity were considered as potent hits of GluN1/GluN3A receptors. **(D)** Representative fluorescence signal of a hit in the FDSS/μCell. The well-to-well variation was 0.01 at 10 μM and 0.02 at 30 μM (calculated with standard deviation). **(E)** Chemical structures of previously reported (black) and newly discovered (red) modulators.

Taking advantage of the established HTS method, we carried out two rounds of screening (Figure 2A). The appropriate concentrations of CGP-78608 were optimized in a preliminary experiment and we found that the saturated concentration of CGP-78608 was approximately 500 nM (Supplementary Figure S1). The tested compounds were incubated for 20 min before the administration of 500 nM CGP-78608. In the first round, a total of 2560 compounds were tested at a fixed dose of 10 μM, and 160 compounds with *F*_drug_/*F*_control_ < 0.75 were defined as the pre-hits (Figure 2B). To improve the accuracy of screening, the pre-hits were further examined at 10 μM and 30 μM in the second round. After the secondary screening, 13 hits were obtained, which met the following criteria: 1) *F*_drug_/*F*_control_ < 0.75 at 10 μM; 2) a dose-dependent change in the Ca^2+^ fluorescence between 10 μM and 30 μM. Notably, although this relatively strict criterion will reduce false positive ratio, it is also possible to miss some potential hits because their effects have been saturated at 10 μM; 3) No effect on NMDARs has been reported previously (Figures 2C,D). As shown in Figure 2D, a substantial fluorescence increase was visible upon CGP-78608 addition in the control condition, which appeared to depend on the concentration of Ca^2+^ and expression of GluN3A (Supplementary Figure S2). The inhibitory effects of these 13 hits were validated at a concentration of 3 μM using whole-cell patch-clamp recording. Among them, 2-chloro-N-[2'-(N-cyanosulfamoyl) biphenyl-4-ylmethyl]-N-(4-methylbenzyl) benzamide (S0859), 7-bromo-2-(4-hydroxyphenyl) benzoxazol-5-ol (WAY200070) and 3-fluoro-1,2-phenylene bis(3-hydroxybenzoate) (WZB117) showed relatively strong inhibitory activity, among which WZB117 was the most potent one (Table 1; Figure 2E). Notably, both the positive control (5,7-DCKA) and four previously reported NMDAR modulators, including L-701324 (Priestley et al., 1996), flupirtine maleate (Osborne et al., 1998; Kornhuber et al., 1999), riluzole (Debono et al., 1993) and D-serine (Wolosker, 2007), were also identified in both the primary screening and secondary screening, suggesting that the HTS method is relatively reliable. Together, our results showed that the HTS method enables the identification of potential modulators targeting GluN1/GluN3A receptors.

**TABLE 1:** Inhibitory effects of 13 compounds on GluN1/GluN3A receptors.

Compound	*I*_drug_/*I*_control_	*n*	Compound	*I*_drug_/*I*_control_	*n*
DMSO	0.94 ± 0.01	3	Rhein	0.80 ± 0.04	4
Avasimibe	0.86 ± 0.03	3	S0859	0.48 ± 0.02	6
GSK2334470	0.84 ± 0.04	6	TAK285	0.92 ± 0.06	3
IC261	0.88 ± 0.01	3	Tirapazamine	1.03 ± 0.05	3
KN93 Phosphate	0.90 ± 0.02	6	WAY200070	0.53 ± 0.02	3
Lonafarnib	0.98 ± 0.02	3	WZB117	0.08 ± 0.02	9
NH125	0.84 ± 0.03	3	SU6668	0.89 ± 0.04	3

Data are shown as the mean ± SEM. All recordings were performed on GluN1/GluN3A Flp-in T-REx 293 stable cell lines. The order of drug application was the same as in FDSS/μCell Ca^2+^ imaging, and the representative current traces are given in Supplementary Figure S4.

### WZB117 Selectively Inhibits the Activity of GluN1/GluN3 Receptors

**FIGURE 3:**
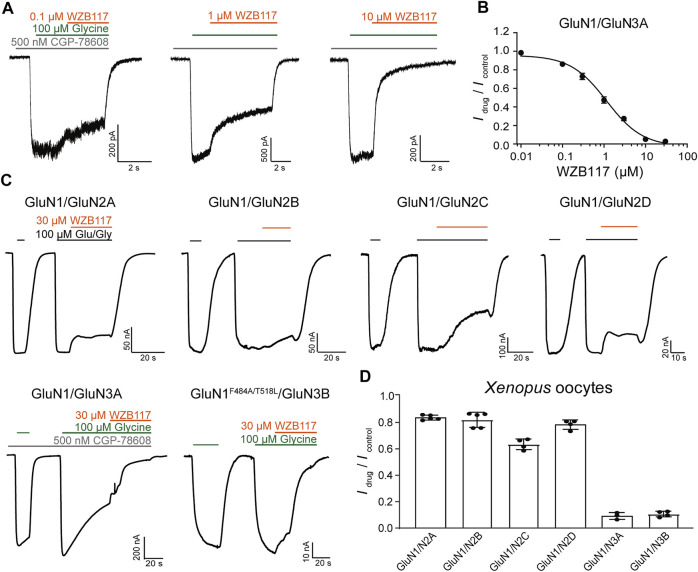
Effects of WZB117 on GluN1/GluN3A receptors. **(A)** Representative traces of GluN1/GluN3A receptors with WZB117 application at the indicated concentrations. Whole-cell patch-clamp recording was performed on HEK-293 cells stably expressing GluN1/GluN3A receptors. **(B)** Dose-response curve of WZB117 on recombinant GluN1/GluN3A receptors (IC_50_ = 1.15 ± 0.34 μM, *n* = 6-7). **(C)** Representative traces of indicated receptors with WZB117 application. TEVC recording was performed on *Xenopus* oocyte expressing indicated receptors. Since currents were not observed for WT GluN1/GluN3B receptors, the mutant channel GluN1-4a^F484A/T518L^/GluN3B was used instead (Kvist et al., 2013). GluN1-1a was always used unless otherwise stated. **(D)** Inhibitory effects of WZB117 on various NMDAR subtypes in *Xenopus* oocytes.

To fully understand the influences of WZB117 on NMDA receptors, the inhibitory activity and subtype selectivity of WZB117 were evaluated in HEK-293 cells stably expressing GluN1/GluN3A receptors and *Xenopus* oocytes transiently expressing NMDARs, respectively. As shown in Figure 3A, WZB117 dose-dependently suppressed the GluN1/GluN3A receptor-mediated currents in the presence of 100 μM glycine. The inhibitory data were fitted to the Hill equation, revealing an IC_50_ value of 1.15 ± 0.34 µM (Figure 3B). The selectivity of WZB117 against other NMDARs was examined in *Xenopus* oocytes. Consistent with the inhibitory effect observed in the GluN1/GluN3A stable cell line, 30 μM WZB117 potently suppressed the currents recorded in the *Xenopus* oocytes expressing GluN1/GluN3A receptors (Figures 3C,D). Due to the failure to record currents of WT GluN1/GluN3B receptors, GluN1-4a^F484A/T518L^/GluN3B mutants were used here, which had been reported to delay desensitization and amplify the currents (Kvist et al., 2013). We found that 30 μM WZB117 showed inhibitory effects on the GluN1-4a^F484A/T518L^/GluN3B receptor-mediated currents similar to those on the GluN1/GluN3A receptors-mediated currents (Figures 3C,D). To induce the GluN1/GluN2 (2A-2D) receptors-mediated currents, 100 μM glycine and 100 μM glutamate were coapplied. Infusion of 30 μM WZB117 produced relatively weak inhibition on the GluN1/GluN2 (2A-2D) receptors (Figures 3C,D). Our results indicated that WZB117 is a relatively selective inhibitor of GluN1/GluN3 receptors.

### WZB117 Inhibits GluN3-NMDARs Neuronal Responses

**FIGURE 4:**
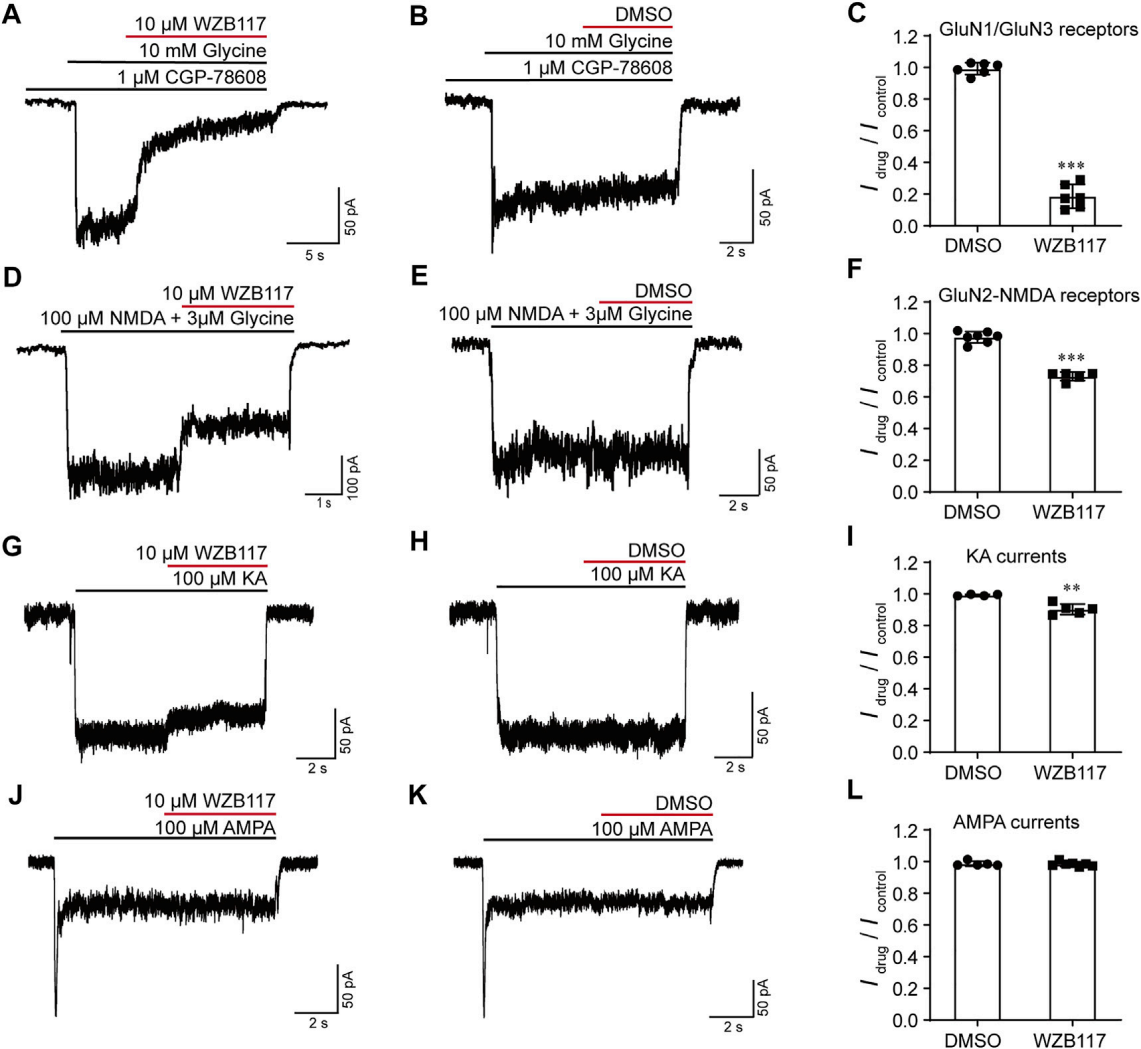
Effects of WZB117 on the native iGluRs. **(A–C)** WZB117 (10 μM) potently inhibited GluN1/GluN3 receptors on acutely isolated rat hippocampal neurons. **(D–L)** WZB117 (10 μM) showed relatively weak inhibition of GluN2-NMDARs **(D–F)** and non-NMDA receptors **(G–L)**. Significance was tested using a t-test; ∗*p* < 0.05, ∗∗*p* < 0.01 and ∗∗∗*p* < 0.001.

The potent and selective inhibition of WZB117 on GluN1/GluN3 receptors expressed in the heterologous system prompted us to study whether WZB117 could inhibit native GluN1/GluN3 receptors in juvenile hippocampal neurons (Grand et al., 2018; Zhu et al., 2020). Consistent with the results obtained in HEK-293 cells stably expressing GluN1/GluN3A receptors, after incubation with CGP-78608, perfusion of 10 μM WZB117 produced a strong inhibition (*I*_drug_/*I*_control_ = 0.18 ± 0.03) of glycine-induced currents in neurons (Figures 4A–C). In contrast, administration of 10 μM WZB117 showed a relatively weak inhibition (*I*_drug_/*I*_control_ = 0.73 ± 0.01) on the native currents primarily mediated by GluN2-containing NMDARs (Figures 4D–F). However, this inhibitory effect was still larger than that of 30 μM WZB117 on the recombinant GluN1/GluN2 receptors on *Xenopus* oocytes (Figures 3C,D), suggesting that the native currents are possibly not only derived from GluN1/GluN2 receptors, but are also partially originated from GluN1/GluN2/GluN3 receptors. This speculation was then examined in the HEK-293 cells transiently transfected with GluN1/GluN2/GluN3 (1:1:2 ratio) plasmids. We found that the inhibitory effect of 30 μM WZB117 on GluN1/GluN2A/GluN3A (*I*_drug_/*I*_cotrol_ = 0.22 ± 0.04) was indeed higher than those of GluN1/GluN2A (*I*_drug_/*I*_cotrol_ = 0.84 ± 0.01) (Supplementary Figure S3). Notably, when the three plasmids were expressed simultaneously, there was a mixture of GluN1/GluN2A, GluN1/GluN3A and GluN1/GluN2A/GluN3A receptors on the plasma membranes, making it difficult to conclude the real effects on GluN1/GluN2A/GluN3A receptors. AMPA and KA receptors are also important iGluRs expressed in the central nervous system. We found that, at 10 μM, WZB117 showed little effect on the KA-induced currents (Figures 4G–I) and was almost ineffective on the AMPA -induced currents (Figures 4J–L). Our data revealed that WZB117 preferentially suppresses native GluN1/GluN3 currents over those of the other iGluRs.

### WZB117 is a Negative Allosteric Modulator of GluN1/GluN3A Receptors

**FIGURE 5:**
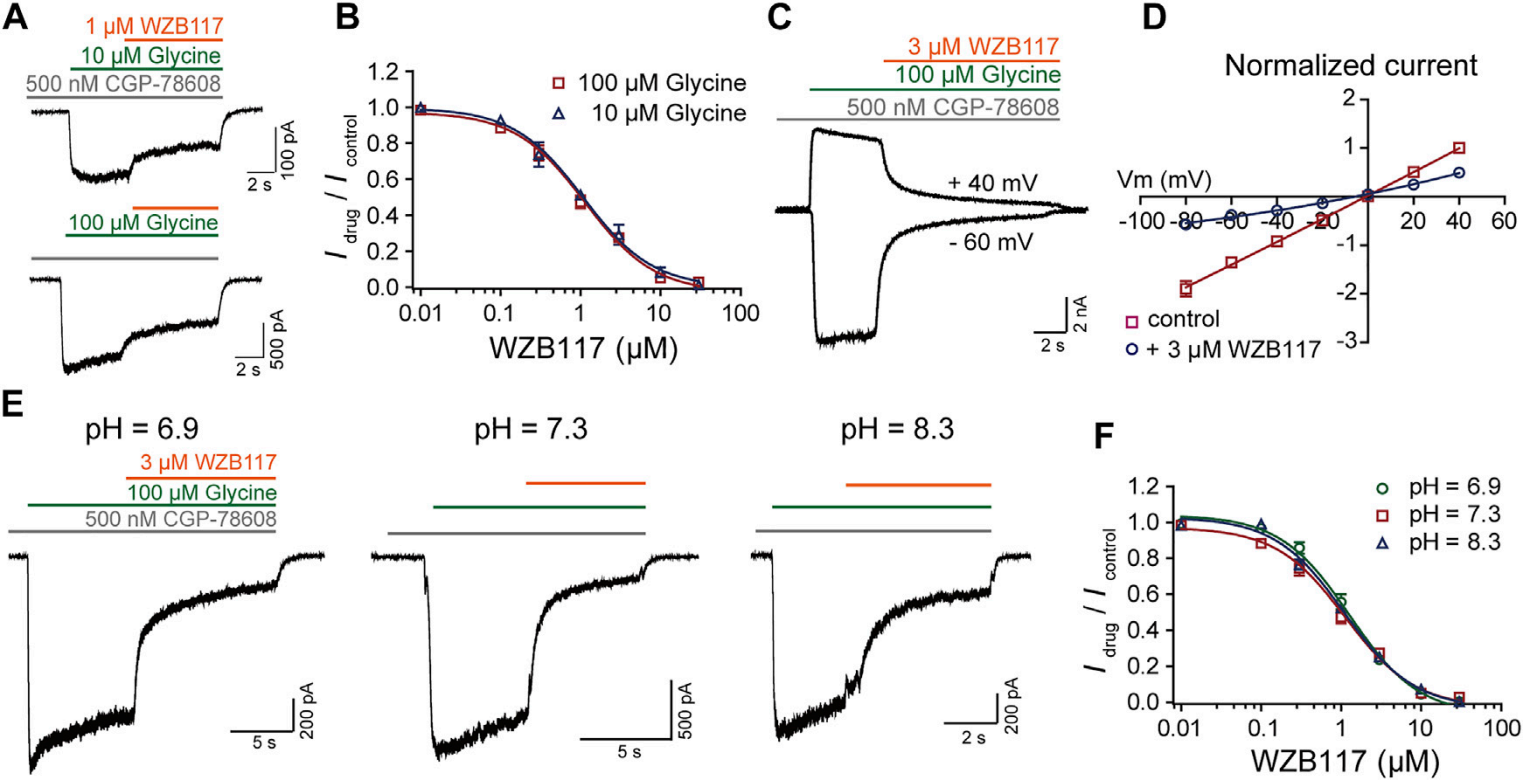
Inhibition mechanism of WZB117 on GluN1/GluN3A receptors. **(A)** Representative traces of GluN1/GluN3A receptors in the presence of glycine at the indicated concentrations. **(B)** Dose-response curves of WZB117 under agonistic conditions of 10 μM (blue) and 100 μM (red) glycine. **(C)** Representative traces of GluN1/GluN3A receptors in the presence of 3 μM WZB117 at membrane potentials of −60 mV and +40 mV. **(D)** Effect of 3 μM WZB117 on the current-voltage relationship of GluN1/GluN3A receptors. The *I*-*V* curves were recorded at membrane potentials ranging from −80 mV to +40 mV, and the current amplitudes were normalized to the response of control at +40 mV. **(E)** Representative traces following 3 μM WZB117 inhibition of GluN1/GluN3A receptors at pH values of 6.9, 7.3, and 8.3. **(F)** Dose-response curves of WZB117 on GluN1/GluN3A receptors at pH of 6.9 (green), 7.3 (red) and 8.3 (blue). All data were recorded from GluN1/GluN3A Flp-in T-REx 293 stable cell lines.

To understand how WZB117 inhibits GluN1/GluN3A receptors, the dose-response curves of WZB117 were compared in the presence of 10 μM and 100 μM glycine individually. The similar IC_50_ values indicated that WZB117 is not a competitive antagonist for the glycine binding sites (IC_50_ = 1.15 ± 0.34 μM at 100 μM glycine; IC_50_ = 1.08 ± 0.31 μM at 10 μM glycine) (Figure 5B). The voltage-dependence of WZB117 was also evaluated. As shown in Figure 5C, there was no significant change of inhibition activity at +40 mV and −60 mV. The current-voltage relationship (*I*-*V* curve) of WZB117 also argued against the possibility that WZB117 is a voltage-dependent pore blocker (Figure 5D). Given that extracellular pH conditions can affect the current amplitudes of GluN1/GluN3 receptors (Cummings and Popescu, 2016), we generated dose-response curves for WZB117 at pH values of 6.9, 7.3, and 8.3, respectively. The comparable IC_50_ values under different pH conditions indicated that WZB117 is a pH-independent antagonist (IC_50_ = 1.27 ± 0.39 μM at pH 6.9; IC_50_ = 1.15 ± 0.34 μM at pH 7.3; IC_50_ = 1.09 ± 0.22 μM at pH 8.3) (Figures 5E,F). Together, our data showed that WZB117 is a negative allosteric modulator which inhibits GluN1/GluN3A receptors in a glycine-, voltage- and pH-independent manner.

### Molecular Determinants of WZB117 Modulation

**FIGURE 6:**
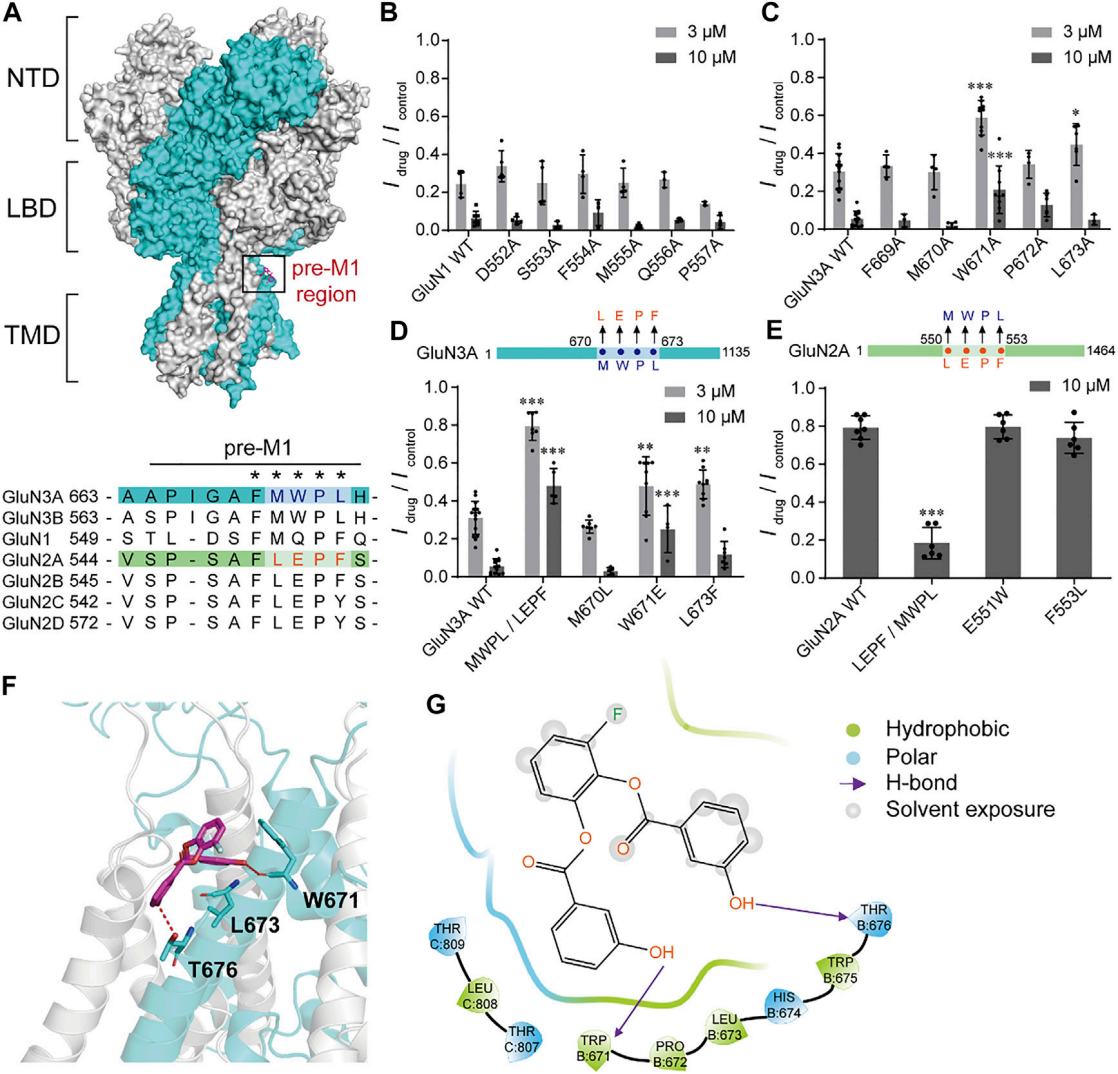
Critical residues for WZB117 inhibition. **(A)** Homology model of the GluN1/GluN3A structure based on the structures of GluN1/GluN2B receptor (PDB ID: 6WHS) and the LBD of GluN3A subunit (PDB ID: 2RC7). GluN1 and GluN3A subunits are shown in gray and cyan, respectively (NTD: the N-terminal domain; LBD: the ligand binding domain; TMD: the transmembrane domain). The black box indicates the pre-M1 region. The bottom panel shows the sequence alignment of pre-M1 in all NMDAR subunits. Residues marked with an asterisk were selected for mutagenesis and whole-cell patch-clamp recordings. **(B and C)** Scanning mutagenesis across the pre-M1 region of GluN1 **(B)** and GluN3A **(C)** subunits. **(D and E)** Inhibition of WZB117 after amino acid substitutions in the pre-M1 regions between GluN3A **(D)** and GluN2A **(E)** subunits. **(F)** Putative binding mode of WZB117. The compound is shown as magenta sticks, and key residues are shown as cyan sticks. Hydrogen bonds are shown as a red dashed line. **(G)** 2D diagram of the interactions between compounds WZB117 and GluN1/GluN3A receptors. All recordings were carried out in transiently transfected CHO cells. Significance was tested using one-way ANOVA; ∗*p* < 0.05, ∗∗*p* < 0.01 and ∗∗∗*p* < 0.001.

To explore the potential action sites of WZB117, we investigated whether the pre-M1 region, previously known to be critical for other inhibitors such as EU1180-438 (Zhu et al., 2020), contributes to WZB117 inhibition. Pre-M1 is a helix connecting the LBD and M1 with poor homology between GluN3 and other subunits of NMDARs (Figure 6A). Alanine-scanning mutagenesis across the pre-M1 region of GluN1 and GluN3A subunits was performed individually. We found that the mutations W671A and L673A of the GluN3A subunit significantly influenced the inhibitory effect of WZB117 (Figure 6C), while none of the mutations in the pre-M1 region of the GluN1 subunit showed a detectable influence on WZB117 activity (Figure 6B), which preliminarily indicated that GluN3A might be the dominant subunit for WZB117 activity. Then, we generated two chimeras of GluN3A^670-673 MWPL/LEPF^ and GluN2A^550-553 LEPF/MWPL^ based on the distinct effects of WZB117 between GluN1/GluN3A and GluN1/GluN2A (Figures 3C,D). Interestingly, after swapping the four amino acids in the pre-M1 regions of GluN2A and GluN3A, the chimera GluN1/GluN2A^550-553 LEPF/MWPL^ acquired higher sensitivity to WZB117 (Figure 6E), while the inhibition on the chimera GluN3A^670-673 MWPL/LEPF^ was greatly reduced (Figure 6D), which validated that pre-M1 of GluN3A is a key region for the inhibitory effect of WZB117.

### *In Silico* Modeling of WZB117 Binding Sites

We have shown that the pre-M1 region in GluN3A contributes to WZB117 inhibition. Then WZB117 was docked into the potential binding pocket at pre-M1 region based on the homology model of GluN1/GluN3A receptors (Figure 6A). As shown in Supplementary Figure S5A, there are two neighboring cavities in the pre-M1 region. Cavity C1 refers to the potential binding pocket of EU1180-438 (Zhu et al., 2020), while WZB117 might bind to the Cavity C2 since it is surrounded by residues affecting WZB117 inhibition, which are W671 and L673 of GluN3A. For GluN1/GluN2A receptors, there is no obvious pocket located at the corresponding sites (Supplementary Figure S5B), which may account for the lower inhibitory activity of WZB117 against GluN1/GluN2A receptors. The putative binding mode of WZB117 suggested that the compound WZB117 may form hydrogen bonds with W671 as well as hydrophobic interactions with L673 (Figures 6F,G), confirming the important roles of the two residues. As substitutions of the two residues by glutamate, alanine or phenylalanine might lead to conformational changes of the main chain, thereby destroying the hydrogen bond and the hydrophobic interactions, the reduction of the inhibitory efficacy of WZB117 on the corresponding mutants might be ascribed to the destruction of these original interactions. Together, the molecular docking study supported the notion that pre-M1 is a key region for WZB117 activity.

## Discussion

To date, only few GluN3 modulators have been reported (Crawley et al., 2022). Due to the low sequence identity and structural conservation between GluN3 and the other NMDAR subunits, GluN3 subunits are hyposensitive to both competitive modulators acting on GluN2 subunits and broad-spectrum channel blockers (Chatterton et al., 2002; McClymont et al., 2012). GluN3A seems to be an attractive target compared to other NMDAR subunits. First, the expression level of GluN3A was found to be abnormally elevated in pathological conditions while low expression levels were found in healthy adults (Kehoe et al., 2013; Wesseling and Perez-Otano, 2015; Pérez-Otaño et al., 2016). Second, inhibition of GluN3A by genetic intervention at different developmental stages can prevent or rescue kinds of Huntington’s disease symptoms ranging from neuronal survival to cognitive and motor functions in mouse models (Marco et al., 2013; Marco et al., 2018). However, the feasibility of GluN3A as a therapeutic target has not been investigated from the perspective of molecular blockade. Similarly, the physiological roles of GluN3B are unclear due to limited research. Therefore, the discovery of GluN3-specific small-molecule modulators is of great significance.

Here, we established a cell-based calcium assay for screening GluN1/GluN3A modulators. First, the Ca^2+^ fluorescence signal is stable with good temporal resolution and sensitivity, and the screening window can be flexibly optimized by changing the stimulation conditions. Second, comparing to the isolated LBD of GluN3A applied in the screening (Kvist et al., 2013), the full length of GluN1/GluN3A receptors may greatly facilitate the discovery of new molecules with different characteristics. In our study, a total of 2560 compounds were tested, and finally 3 compounds (≥50% inhibition at 3 μM) were identified as GluN1/GluN3A receptor antagonists with completely different structures than those of the previously reported molecules (Figure 2; Table 1). Based on both the positive experiences and negative experiences learned from this pilot screening, a large-scale HTS would accelerate the identification of more GluN3 modulators.

Among the three identified hits in the pilot screening, WZB117 showed strong inhibitory potency with an IC_50_ value of 1.15 ± 0.34 μM (Figure 3), which is similar to that of EU1180-438 (IC_50_ = 1.80 ± 0.30 μM) (Zhu et al., 2020) and much higher than that of TK compounds (Kvist et al., 2013). Due to the desensitization of the GluN1/GluN3 receptors (Grand et al., 2018), the inhibitory effect of WZB117 could be overestimated in the TEVC and whole-cell patch clamp experiments. In addition, WZB117 exhibited a good subtype selectivity for GluN1/GluN3 receptors relative to GluN1/GluN2 receptors and other iGluRs in acutely isolated rat hippocampal neurons (Figure 3; Figure 4). However, to define WZB117 as a good pharmacological tool, a more comprehensive evaluation of selectivity is needed. For example, WZB117 has been reported to be a glucose transporter (GluT) inhibitor with antitumor activity. Besides, S0859 is a high-affinity inhibitor of Na^+^/HCO_3_^−^ cotransporter (NBC) with potential activity in the regulation of cardiac function, and WAY-200070 is an agonist of estrogen receptor beta (ERRβ) with potential anxiolytic and antidepressant activity (Ch’en et al., 2008; Hughes et al., 2008; Liu et al., 2012; Xintaropoulou et al., 2015; Wei et al., 2018). Accordingly, it appears difficult to identify an ideal probe with potent activity and excellent selectivity for GluN3A or GluN3B simply through HTS. Further structural modifications would help to improve both potency and selectivity and eliminate potential side effects of these lead compounds.

In the past 30 years, clinical studies have shown that competitive antagonists and channel blockers of NMDARs commonly display severe side effects, possibly due to their broad-spectrum inhibitory effects, and thereby greatly limiting their clinical development (Kalia et al., 2008; Hardingham and Bading, 2010; Traynelis et al., 2010; Hansen et al., 2012). Allosteric modulators generally show strong subunit-selectivity and better clinical development prospects as they bind to less conserved pockets (Hansen et al., 2012; Zhu and Paoletti, 2015; Burnell et al., 2019; Strong et al., 2021). Our data indicated that WZB117 is a negative allosteric modulator inhibiting GluN1/GluN3A receptors in a glycine-, voltage- and pH-independent manner (Figure 5). We further demonstrated that GluN3A pre-M1 is a potential binding pocket for WZB117, which was profoundly implicated by the significant changes in the activity of WZB117 against the chimeras GluN1/GluN3A^670-673 MWPL/LEPF^ and GluN1/GluN2A^550-553 LEPF/MWPL^. Particularly, the chimera GluN1/GluN2A^550-553 LEPF/MWPL^, owning a short motif containing only four amino acids from the GluN3A pre-M1 region, gained sensitivity towards WZB117 inhibition, which further confirmed the importance of the pre-M1 region (Figure 6). Recent structural pharmacological studies of NMDARs have provided cogent evidence for the important contribution of the linkers between the LBDs and the transmembrane domains (TMDs) to channel gating (Wang et al., 2021). In addition, many *de novo* mutations related to neurological diseases, such as epilepsy and developmental delay, are associated with the pre-M1 regions of both GluN1 and GluN2 subunits, which also appear to affect the kinetics of the channels (Sobolevsky et al., 2007; Ogden et al., 2017; Vyklicky et al., 2018; XiangWei et al., 2019; McDaniel et al., 2020). Although no studies about GluN3A pre-M1 related structures or pathogenic mutations have been reported, our work provides insights from a pharmacological perspective that GluN3A pre-M1 is a potential target for drug development. Considering that the critical residues of WZB117 are different from those of EU1180-438, there may be different drug binding pockets near to each other (Supplementary Figure S5A).

In conclusion, we have established a cell-based high-throughput screening and identified WZB117 as a novel and potent allosteric modulator against GluN1/GluN3 receptors. WZB117 exhibited relative selectivity for GluN1/GluN3 inhibition over other iGluRs. A mechanistic study revealed that multiple residues located at pre-M1 region of the GluN3A subunit are critical for the activity of WZB117. Our study provided new active molecules for channel function research and drug development targeting GluN3-NMDARs.

## Data Availability

The raw data supporting the conclusion of this article will be made available by the authors, without undue reservation.

## References

[B1] Burnell E. S. Irvine M. Fang G. Sapkota K. Jane D. E. Monaghan D. T. (2019). Positive and Negative Allosteric Modulators of N-Methyl-D-Aspartate (NMDA) Receptors: Structure-Activity Relationships and Mechanisms of Action. J. Med. Chem. 62 (1), 3–23. 10.1021/acs.jmedchem.7b01640 29446949 PMC6368479

[B2] Ch'en F. F. Villafuerte F. C. Swietach P. Cobden P. M. Vaughan-Jones R. D. (2008). S0859, an N-Cyanosulphonamide Inhibitor of Sodium-Bicarbonate Cotransport in the Heart. Br. J. Pharmacol. 153 (5), 972–982. 10.1038/sj.bjp.0707667 18204485 PMC2267275

[B3] Chatterton J. E. Awobuluyi M. Premkumar L. S. Takahashi H. Talantova M. Shin Y. (2002). Excitatory glycine Receptors Containing the NR3 Family of NMDA Receptor Subunits. Nature 415 (6873), 793–798. 10.1038/nature715 11823786

[B4] Chou T. H. Tajima N. Romero-Hernandez A. Furukawa H. (2020). Structural Basis of Functional Transitions in Mammalian NMDA Receptors. Cell 182 (2), 357. 10.1016/j.cell.2020.05.052 32610085 PMC8278726

[B5] Ciabarra A. M. Sullivan J. M. Gahn L. G. Pecht G. Heinemann S. Sevarino K. A. (1995). Cloning and Characterization of Chi-1: a Developmentally Regulated Member of a Novel Class of the Ionotropic Glutamate Receptor Family. J. Neurosci. 15 (10), 6498–6508. 10.1523/jneurosci.15-10-06498.1995 7472412 PMC6577996

[B6] Crawley O. Conde-Dusman M. J. Pérez-Otaño I. (2022). GluN3A NMDA Receptor Subunits: More Enigmatic Than Ever? J. Physiol. 600 (2), 261–276. 10.1113/jp280879 33942912

[B7] Cummings K. A. Popescu G. K. (2016). Protons Potentiate GluN1/GluN3A Currents by Attenuating Their Desensitisation. Sci. Rep. 6, 23344. 10.1038/srep23344 27000430 PMC4802338

[B8] Das S. Sasaki Y. F. Rothe T. Premkumar L. S. Takasu M. Crandall J. E. (1998). Increased NMDA Current and Spine Density in Mice Lacking the NMDA Receptor Subunit NR3A. Nature 393 (6683), 377–381. 10.1038/30748 9620802

[B9] Debono M. W. Le Guern J. Canton T. Doble A. Pradier L. (1993). Inhibition by Riluzole of Electrophysiological Responses Mediated by Rat Kainate and NMDA Receptors Expressed in Xenopus Oocytes. Eur. J. Pharmacol. 235 (2-3), 283–289. 10.1016/0014-2999(93)90147-a 7685290

[B10] Fiuza M. González-González I. Pérez-Otaño I. (2013). GluN3A Expression Restricts Spine Maturation via Inhibition of GIT1/Rac1 Signaling. Proc. Natl. Acad. Sci. U. S. A. 110 (51), 20807–20812. 10.1073/pnas.1312211110 24297929 PMC3870762

[B11] Friesner R. A. Banks J. L. Murphy R. B. Halgren T. A. Klicic J. J. Mainz D. T. (2004). Glide: a New Approach for Rapid, Accurate Docking and Scoring. 1. Method and Assessment of Docking Accuracy. J. Med. Chem. 47 (7), 1739–1749. 10.1021/jm0306430 15027865

[B12] Gee K. R. Brown K. A. Chen W. N. Bishop-Stewart J. Gray D. Johnson I. (2000). Chemical and Physiological Characterization of Fluo-4 Ca(2+)-Indicator Dyes. Cell Calcium 27 (2), 97–106. 10.1054/ceca.1999.0095 10756976

[B13] Glantz L. A. Lewis D. A. (2000). Decreased Dendritic Spine Density on Prefrontal Cortical Pyramidal Neurons in Schizophrenia. Arch. Gen. Psychiatry 57 (1), 65–73. 10.1001/archpsyc.57.1.65 10632234

[B14] Grand T. Abi Gerges S. David M. Diana M. A. Paoletti P. (2018). Unmasking GluN1/GluN3A Excitatory glycine NMDA Receptors. Nat. Commun. 9 (1), 4769. 10.1038/s41467-018-07236-4 30425244 PMC6233196

[B15] Hansen K. B. Ogden K. K. Traynelis S. F. (2012). Subunit-selective Allosteric Inhibition of glycine Binding to NMDA Receptors. J. Neurosci. 32 (18), 6197–6208. 10.1523/jneurosci.5757-11.2012 22553026 PMC3355950

[B16] Hansen K. B. Wollmuth L. P. Bowie D. Furukawa H. Menniti F. S. Sobolevsky A. I. (2021). Structure, Function, and Pharmacology of Glutamate Receptor Ion Channels. Pharmacol. Rev. 73 (4), 298–487. 10.1124/pharmrev.120.000131 34753794 PMC8626789

[B17] Hansen K. B. Yi F. Perszyk R. E. Furukawa H. Wollmuth L. P. Gibb A. J. (2018). Structure, Function, and Allosteric Modulation of NMDA Receptors. J. Gen. Physiol. 150 (8), 1081–1105. 10.1085/jgp.201812032 30037851 PMC6080888

[B18] Hardingham G. E. Bading H. (2010). Synaptic versus Extrasynaptic NMDA Receptor Signalling: Implications for Neurodegenerative Disorders. Nat. Rev. Neurosci. 11 (10), 682–696. 10.1038/nrn2911 20842175 PMC2948541

[B19] Hughes Z. A. Liu F. Platt B. J. Dwyer J. M. Pulicicchio C. M. Zhang G. (2008). WAY-200070, a Selective Agonist of Estrogen Receptor Beta as a Potential Novel Anxiolytic/antidepressant Agent. Neuropharmacology 54 (7), 1136–1142. 10.1016/j.neuropharm.2008.03.004 18423777

[B20] Jin Z. Bhandage A. K. Bazov I. Kononenko O. Bakalkin G. Korpi E. R. (2014). Selective Increases of AMPA, NMDA, and Kainate Receptor Subunit mRNAs in the hippocampus and Orbitofrontal Cortex but Not in Prefrontal Cortex of Human Alcoholics. Front. Cell Neurosci. 8, 11. 10.3389/fncel.2014.00011 24523671 PMC3905203

[B21] Kalia L. V. Kalia S. K. Salter M. W. (2008). NMDA Receptors in Clinical Neurology: Excitatory Times Ahead. Lancet Neurol. 7 (8), 742–755. 10.1016/s1474-4422(08)70165-0 18635022 PMC3589564

[B22] Karakas E. Furukawa H. (2014). Crystal Structure of a Heterotetrameric NMDA Receptor Ion Channel. Science 344 (6187), 992–997. 10.1126/science.1251915 24876489 PMC4113085

[B23] Kehoe L. A. Bellone C. De Roo M. Zandueta A. Dey P. N. Pérez-Otaño I. (2014). GluN3A Promotes Dendritic Spine Pruning and Destabilization during Postnatal Development. J. Neurosci. 34 (28), 9213–9221. 10.1523/JNEUROSCI.5183-13.2014 25009255 PMC6608362

[B24] Kehoe L. A. Bernardinelli Y. Muller D. (2013). GluN3A: an NMDA Receptor Subunit with Exquisite Properties and Functions. Neural Plast. 2013, 145387. 10.1155/2013/145387 24386575 PMC3872238

[B25] Kornhuber J. Bleich S. Wiltfang J. Maler M. Parsons C. G. (1999). Flupirtine Shows Functional NMDA Receptor Antagonism by Enhancing Mg2+ Block via Activation of Voltage Independent Potassium Channels. Rapid Communication. J. Neural Transm. (Vienna) 106 (9-10), 857–867. 10.1007/s007020050206 10599868

[B26] Kvist T. Greenwood J. R. Hansen K. B. Traynelis S. F. Bräuner-Osborne H. (2013). Structure-based Discovery of Antagonists for GluN3-Containing N-Methyl-D-Aspartate Receptors. Neuropharmacology 75, 324–336. 10.1016/j.neuropharm.2013.08.003 23973313 PMC3865070

[B27] Le Guilloux V. Schmidtke P. Tuffery P. (2009). Fpocket: an Open Source Platform for Ligand Pocket Detection. BMC Bioinforma. 10, 168. 10.1186/1471-2105-10-168 PMC2700099 19486540

[B28] Liu Y. Cao Y. Zhang W. Bergmeier S. Qian Y. Akbar H. (2012). A Small-Molecule Inhibitor of Glucose Transporter 1 Downregulates Glycolysis, Induces Cell-Cycle Arrest, and Inhibits Cancer Cell Growth *In Vitro* and *In Vivo* . Mol. Cancer Ther. 11 (8), 1672–1682. 10.1158/1535-7163.Mct-12-0131 22689530

[B29] Mahfooz K. Marco S. Martínez-Turrillas R. Raja M. K. Pérez-Otaño I. Wesseling J. F. (2016). GluN3A Promotes NMDA Spiking by Enhancing Synaptic Transmission in Huntington's Disease Models. Neurobiol. Dis. 93, 47–56. 10.1016/j.nbd.2016.04.001 27072890

[B30] Marco S. Giralt A. Petrovic M. M. Pouladi M. A. Martínez-Turrillas R. Martínez-Hernández J. (2013). Suppressing Aberrant GluN3A Expression Rescues Synaptic and Behavioral Impairments in Huntington's Disease Models. Nat. Med. 19 (8), 1030–1038. 10.1038/nm.3246 23852340 PMC3936794

[B31] Marco S. Murillo A. Pérez-Otaño I. (2018). RNAi-Based GluN3A Silencing Prevents and Reverses Disease Phenotypes Induced by Mutant Huntingtin. Mol. Ther. 26 (8), 1965–1972. 10.1016/j.ymthe.2018.05.013 29914757 PMC6094357

[B32] McClymont D. W. Harris J. Mellor I. R. (2012). Open-channel Blockade Is Less Effective on GluN3B Than GluN3A Subunit-Containing NMDA Receptors. Eur. J. Pharmacol. 686 (1-3), 22–31. 10.1016/j.ejphar.2012.04.036 22564863 PMC3657159

[B33] McDaniel M. J. Ogden K. K. Kell S. A. Burger P. B. Liotta D. C. Traynelis S. F. (2020). NMDA Receptor Channel Gating Control by the Pre-M1 Helix. J. Gen. Physiol. 152 (4), e201912362. 10.1085/jgp.201912362 32221541 PMC7141592

[B34] Mueller H. T. Meador-Woodruff J. H. (2004). NR3A NMDA Receptor Subunit mRNA Expression in Schizophrenia, Depression and Bipolar Disorder. Schizophr. Res. 71 (2-3), 361–370. 10.1016/j.schres.2004.02.016 15474907

[B35] Ogden K. K. Chen W. Swanger S. A. McDaniel M. J. Fan L. Z. Hu C. (2017). Molecular Mechanism of Disease-Associated Mutations in the Pre-M1 Helix of NMDA Receptors and Potential Rescue Pharmacology. PLoS Genet. 13 (1), e1006536. 10.1371/journal.pgen.1006536 28095420 PMC5240934

[B36] Osborne N. N. Cazevieille C. Wood J. P. Nash M. S. Pergande G. Block F. (1998). Flupirtine, a Nonopioid Centrally Acting Analgesic, Acts as an NMDA Antagonist. Gen. Pharmacol. 30 (3), 255–263. 10.1016/s0306-3623(97)00355-8 9510072

[B37] Otsu Y. Darcq E. Pietrajtis K. Mátyás F. Schwartz E. Bessaih T. (2019). Control of Aversion by Glycine-Gated GluN1/GluN3A NMDA Receptors in the Adult Medial Habenula. Science 366 (6462), 250–254. 10.1126/science.aax1522 31601771 PMC7556698

[B38] Paoletti P. Bellone C. Zhou Q. (2013). NMDA Receptor Subunit Diversity: Impact on Receptor Properties, Synaptic Plasticity and Disease. Nat. Rev. Neurosci. 14 (6), 383–400. 10.1038/nrn3504 23686171

[B39] Pérez-Otaño I. Larsen R. S. Wesseling J. F. (2016). Emerging Roles of GluN3-Containing NMDA Receptors in the CNS. Nat. Rev. Neurosci. 17 (10), 623–635. 10.1038/nrn.2016.92 27558536

[B40] Pérez-Otaño I. Luján R. Tavalin S. J. Plomann M. Modregger J. Liu X. B. (2006). Endocytosis and Synaptic Removal of NR3A-Containing NMDA Receptors by PACSIN1/syndapin1. Nat. Neurosci. 9 (5), 611–621. 10.1038/nn1680 16617342 PMC1892311

[B41] Perez-Otano I. Schulteis C. T. Contractor A. Lipton S. A. Trimmer J. S. Sucher N. J. (2001). Assembly with the NR1 Subunit Is Required for Surface Expression of NR3A-Containing NMDA Receptors. J. Neurosci. 21 (4), 1228–1237. 10.1523/jneurosci.21-04-01228.2001 11160393 PMC6762235

[B42] Priestley T. Laughton P. Macaulay A. J. Hill R. G. Kemp J. A. (1996). Electrophysiological Characterisation of the Antagonist Properties of Two Novel NMDA Receptor glycine Site Antagonists, L-695,902 and L-701,324. Neuropharmacology 35 (11), 1573–1581. 10.1016/s0028-3908(96)00141-4 9025105

[B43] Roberts A. C. Díez-García J. Rodriguiz R. M. López I. P. Luján R. Martínez-Turrillas R. (2009). Downregulation of NR3A-Containing NMDARs Is Required for Synapse Maturation and Memory Consolidation. Neuron 63 (3), 342–356. 10.1016/j.neuron.2009.06.016 19679074 PMC3448958

[B44] Sasaki Y. F. Rothe T. Premkumar L. S. Das S. Cui J. Talantova M. V. (2002). Characterization and Comparison of the NR3A Subunit of the NMDA Receptor in Recombinant Systems and Primary Cortical Neurons. J. Neurophysiol. 87 (4), 2052–2063. 10.1152/jn.00531.2001 11929923

[B45] Shelley J. C. Cholleti A. Frye L. L. Greenwood J. R. Timlin M. R. Uchimaya M. (2007). Epik: a Software Program for pK( a ) Prediction and Protonation State Generation for Drug-like Molecules. J. Comput. Aided Mol. Des. 21 (12), 681–691. 10.1007/s10822-007-9133-z 17899391

[B46] Sobolevsky A. I. Prodromou M. L. Yelshansky M. V. Wollmuth L. P. (2007). Subunit-specific Contribution of Pore-Forming Domains to NMDA Receptor Channel Structure and Gating. J. Gen. Physiol. 129 (6), 509–525. 10.1085/jgp.200609718 17504910 PMC2151626

[B47] Strong K. L. Epplin M. P. Ogden K. K. Burger P. B. Kaiser T. M. Wilding T. J. (2021). Distinct GluN1 and GluN2 Structural Determinants for Subunit-Selective Positive Allosteric Modulation of N-Methyl-D-Aspartate Receptors. ACS Chem. Neurosci. 12 (1), 79–98. 10.1021/acschemneuro.0c00561 33326224 PMC7967294

[B48] Sucher N. J. Akbarian S. Chi C. L. Leclerc C. L. Awobuluyi M. Deitcher D. L. (1995). Developmental and Regional Expression Pattern of a Novel NMDA Receptor-like Subunit (NMDAR-L) in the Rodent Brain. J. Neurosci. 15 (10), 6509–6520. 10.1523/jneurosci.15-10-06509.1995 7472413 PMC6578025

[B49] Takata A. Iwayama Y. Fukuo Y. Ikeda M. Okochi T. Maekawa M. (2013). A Population-specific Uncommon Variant in GRIN3A Associated with Schizophrenia. Biol. Psychiatry 73 (6), 532–539. 10.1016/j.biopsych.2012.10.024 23237318

[B50] Traynelis S. F. Wollmuth L. P. McBain C. J. Menniti F. S. Vance K. M. Ogden K. K. (2010). Glutamate Receptor Ion Channels: Structure, Regulation, and Function. Pharmacol. Rev. 62 (3), 405–496. 10.1124/pr.109.002451 20716669 PMC2964903

[B51] Ulbrich M. H. Isacoff E. Y. (2007). Subunit Counting in Membrane-Bound Proteins. Nat. Methods 4 (4), 319–321. 10.1038/nmeth1024 17369835 PMC2744285

[B52] von Boehmer L. Liu C. Ackerman S. Gitlin A. D. Wang Q. Gazumyan A. (2016). Sequencing and Cloning of Antigen-specific Antibodies from Mouse Memory B Cells. Nat. Protoc. 11 (10), 1908–1923. 10.1038/nprot.2016.102 27658009

[B53] Vyklicky V. Krausova B. Cerny J. Ladislav M. Smejkalova T. Kysilov B. (2018). Surface Expression, Function, and Pharmacology of Disease-Associated Mutations in the Membrane Domain of the Human GluN2B Subunit. Front. Mol. Neurosci. 11, 110. 10.3389/fnmol.2018.00110 29681796 PMC5897658

[B54] Wang H. Lv S. Stroebel D. Zhang J. Pan Y. Huang X. (2021). Gating Mechanism and a Modulatory Niche of Human GluN1-GluN2A NMDA Receptors. Neuron 109 (15), 2443. 10.1016/j.neuron.2021.05.031 34186027

[B55] Webb B. Sali A. (2016). Comparative Protein Structure Modeling Using MODELLER. Curr. Protoc. Protein Sci. 86, 21–375. 10.1002/cpbi.3 27801516

[B56] Wei M. Lu L. Sui W. Liu Y. Shi X. Lv L. (2018). Inhibition of GLUTs by WZB117 Mediates Apoptosis in Blood-Stage Plasmodium Parasites by Breaking Redox Balance. Biochem. Biophys. Res. Commun. 503 (2), 1154–1159. 10.1016/j.bbrc.2018.06.134 29953861

[B57] Wesseling J. F. Pérez-Otaño I. (2015). Modulation of GluN3A Expression in Huntington Disease: a New N-Methyl-D-Aspartate Receptor-Based Therapeutic Approach? JAMA Neurol. 72 (4), 468–473. 10.1001/jamaneurol.2014.3953 25686081

[B58] Wolosker H. (2007). NMDA Receptor Regulation by D-Serine: New Findings and Perspectives. Mol. Neurobiol. 36 (2), 152–164. 10.1007/s12035-007-0038-6 17952659

[B59] Wong H. K. Liu X. B. Matos M. F. Chan S. F. Pérez-Otaño I. Boysen M. (2002). Temporal and Regional Expression of NMDA Receptor Subunit NR3A in the Mammalian Brain. J. Comp. Neurol. 450 (4), 303–317. 10.1002/cne.10314 12209845

[B60] XiangWei W. Kannan V. Xu Y. Kosobucki G. J. Schulien A. J. Kusumoto H. (2019). Heterogeneous Clinical and Functional Features of GRIN2D-Related Developmental and Epileptic Encephalopathy. Brain 142 (10), 3009–3027. 10.1093/brain/awz232 31504254 PMC6763743

[B61] Xie Z. Q. Tian X. T. Zheng Y. M. Zhan L. Chen X. Q. Xin X. M. (2020). Antiepileptic Geissoschizine Methyl Ether Is an Inhibitor of Multiple Neuronal Channels. Acta Pharmacol. Sin. 41 (5), 629–637. 10.1038/s41401-019-0327-4 31911638 PMC7471432

[B62] Xintaropoulou C. Ward C. Wise A. Marston H. Turnbull A. Langdon S. P. (2015). A Comparative Analysis of Inhibitors of the Glycolysis Pathway in Breast and Ovarian Cancer Cell Line Models. Oncotarget 6 (28), 25677–25695. 10.18632/oncotarget.4499 26259240 PMC4694858

[B63] Yao Y. Harrison C. B. Freddolino P. L. Schulten K. Mayer M. L. (2008). Molecular Mechanism of Ligand Recognition by NR3 Subtype Glutamate Receptors. Embo J. 27 (15), 2158–2170. 10.1038/emboj.2008.140 18636091 PMC2516888

[B64] Yao Y. Mayer M. L. (2006). Characterization of a Soluble Ligand Binding Domain of the NMDA Receptor Regulatory Subunit NR3A. J. Neurosci. 26 (17), 4559–4566. 10.1523/jneurosci.0560-06.2006 16641235 PMC6674067

[B65] Yuan T. Bellone C. (2013). Glutamatergic Receptors at Developing Synapses: the Role of GluN3A-Containing NMDA Receptors and GluA2-Lacking AMPA Receptors. Eur. J. Pharmacol. 719 (1-3), 107–111. 10.1016/j.ejphar.2013.04.056 23872415

[B66] Yuan T. Mameli M. O'Connor E. C. O' Connor E. C. Dey P. N. Verpelli C. (2013). Expression of Cocaine-Evoked Synaptic Plasticity by GluN3A-Containing NMDA Receptors. Neuron 80 (4), 1025–1038. 10.1016/j.neuron.2013.07.050 24183704

[B67] Zhu S. Paoletti P. (2015). Allosteric Modulators of NMDA Receptors: Multiple Sites and Mechanisms. Curr. Opin. Pharmacol. 20, 14–23. 10.1016/j.coph.2014.10.009 25462287

[B68] Zhu Z. Yi F. Epplin M. P. Liu D. Summer S. L. Mizu R. (2020). Negative Allosteric Modulation of GluN1/GluN3 NMDA Receptors. Neuropharmacology 176, 108117. 10.1016/j.neuropharm.2020.108117 32389749 PMC7530031

