# Supplementary material for: Identification of a Subtype-Selective Allosteric Inhibitor of GluN1/GluN3 NMDA Receptors
Source: Front Pharmacol. 2022 Jun 9;13:888308. doi: 10.3389/fphar.2022.888308 (PMC9218946; doi:10.3389/fphar.2022.888308)
Supplement: Supplementary file 1 [file DataSheet1.docx]

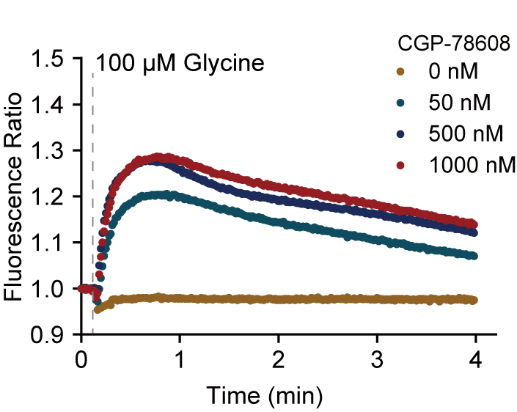


**Supplementary Figure S1** Optimization of CGP-78608 concentrations. The fluorescence signals were recorded in HEK-293 cells stably expressing GluN1/GluN3A receptors using FDSS/μCell. Increased concentrations of CGP-78608 (0-1000 nM) were pre-incubated 20 min before treatment with 100 μM glycine. The data showed that CGP-78608 is necessary for the generation of a fluorescence signal. The saturated concentration of CGP-78608 was approximately 500 nM.


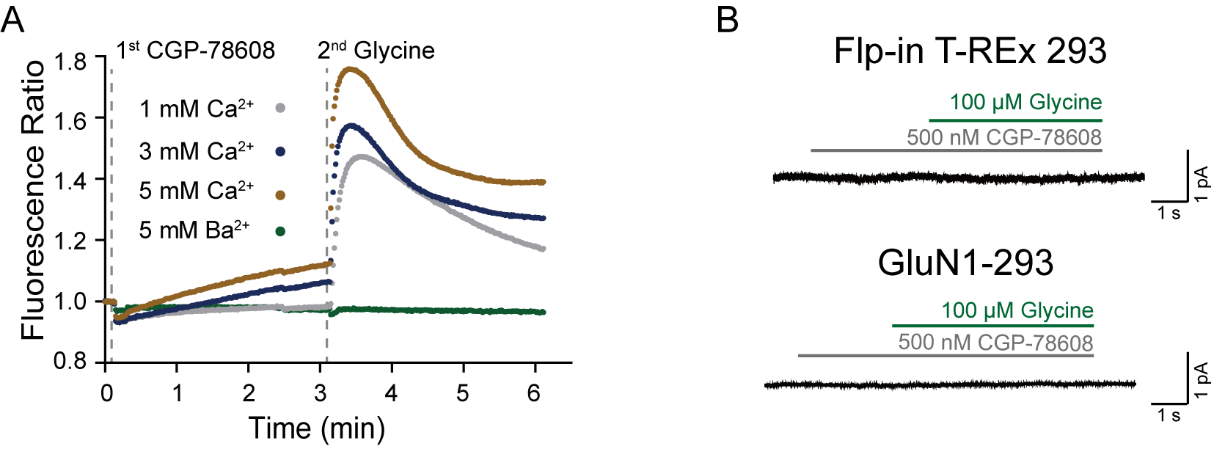


**Supplementary Figure S2** The fluorescence increase upon CGP-78608 addition was related to Ca^2+^ and the expression of GluN3A. **(A)** The fluorescence increase is Ca^2+^-dependent. In HEK-293 cells stably expressing GluN1/GluN3A receptors, application of CGP-78608 induced a more significant “run up” when the extracellular Ca^2+^ concentrations were increased. **(B)** No whole-cell currents were induced by application of CGP-78608 in host cells (Flp-in T-REX 293) and HEK-293 cells stably expressing GluN1 only (GluN1-293). It was consistent to the results in the calcium assays (**Figure 1D**) that unlike in the HEK-293 cells stably expressing GluN1/GluN3A receptors, application of CGP-78608 did not induce “run up” in host cells and GluN1-293, which indicated that the “run up” appeared to be related to the expression of GluN3A subunits. The exact origins of the “run up” warrant further investigations, such as whether it comes from low levels of exogenous glycine.


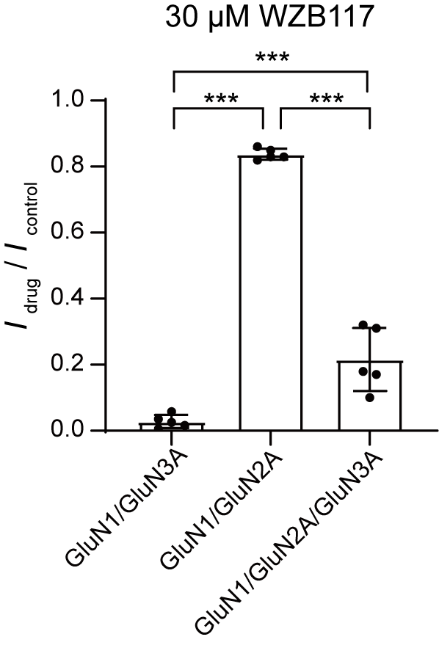


**Supplementary Figure S3** Effects of WZB117 on currents from HEK-293 cells co-expressing GluN1/GluN2A/GluN3A subunits. GluN1/GluN2A/GluN3A receptors were expressed on HEK-293 cells at a ratio of GluN1-EGFP: GluN2A: GluN3A = 1:1:2. The GluN1/GluN2A/GluN3A receptors were stimulated by 100 μM glutamate and 100 μM glycine. The data of GluN1/GluN2A were analyzed from Figure 3D. Significance was tested using one-way ANOVA; **P* < 0.05, ***P* < 0.01 and ****P* < 0.001.


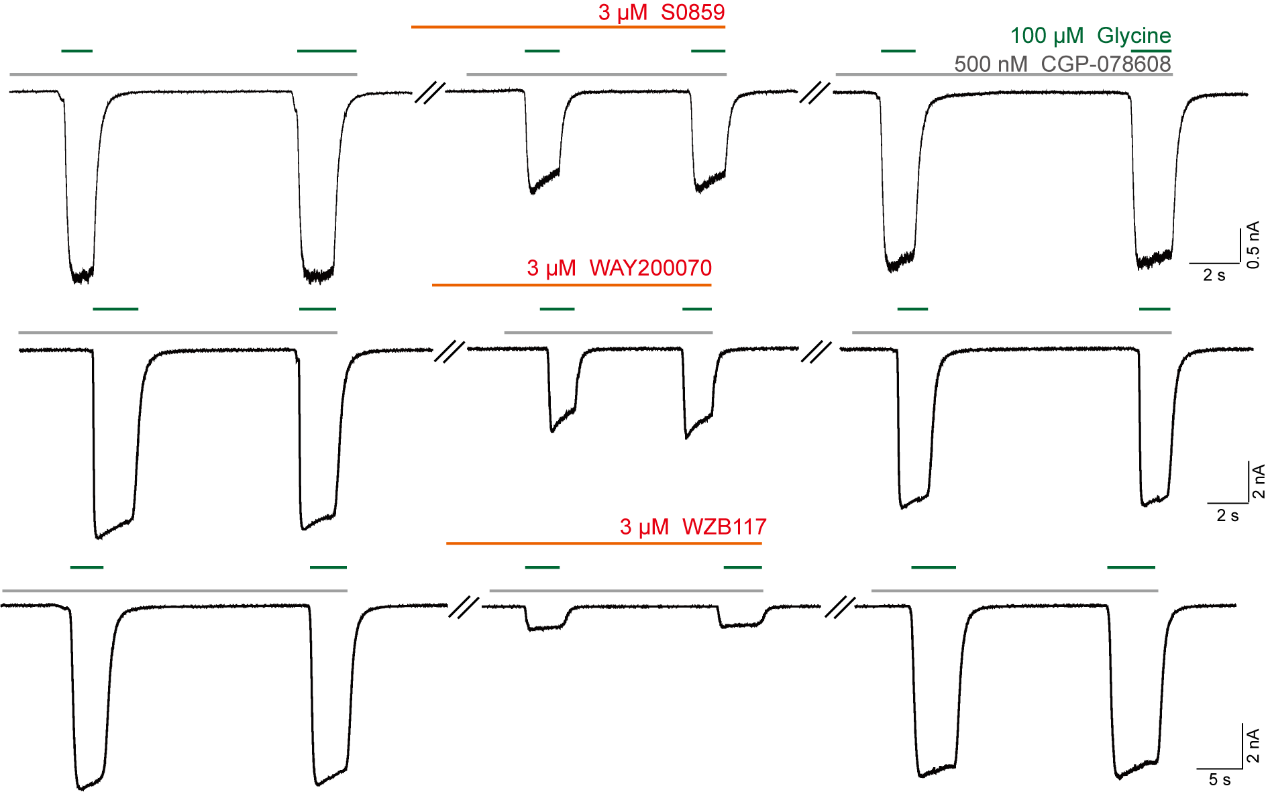
**Supplementary Figure S4** Representative patch-clamp recordings of hits after HTS. Compounds were perfused for 30 s. All data were recorded from GluN1/GluN3A Flp-in T-REx 293 stable cell lines.


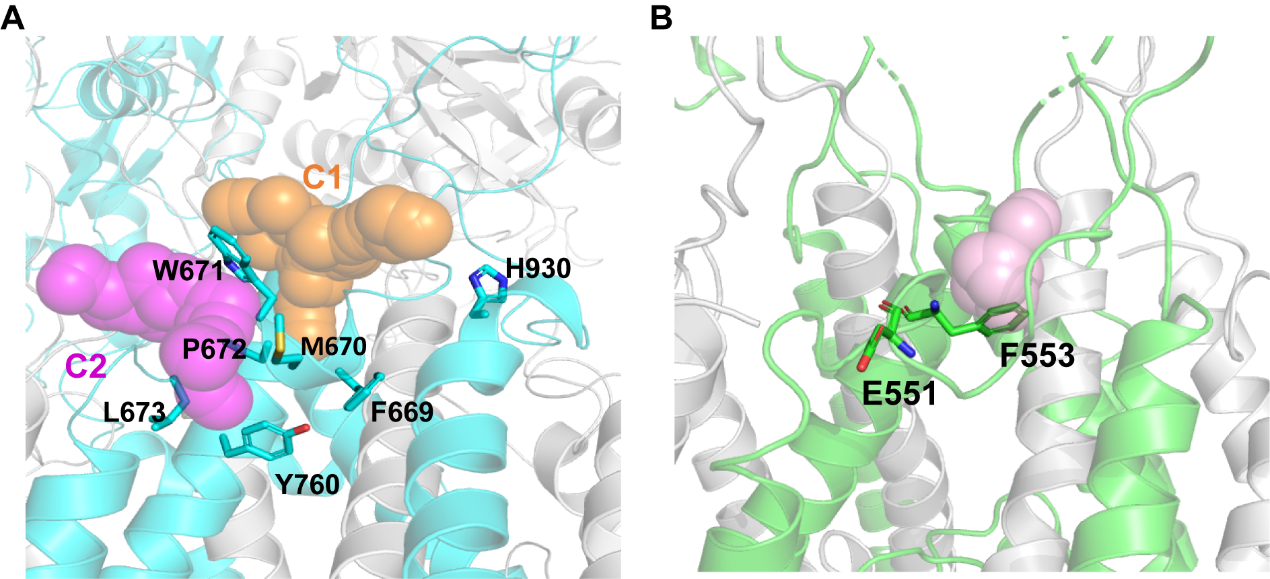


**Supplementary Figure S5** Potential binding mode of WZB117. **(A)** Two potential cavities located at pre-M1 region of GluN3A. **(B)** Potential cavity located at pre-M1 region of GluN2A.
